# Challenges and Solutions in Management of Distal Humerus Fractures

**DOI:** 10.2174/1874325001711011292

**Published:** 2017-11-13

**Authors:** Saif Ul Islam, Alexander William Glover, Mohammad Waseem

**Affiliations:** Macclesfield District General Hospital, Cheshire, United Kingdom

**Keywords:** Humerus, Elbow, Humerus fractures, Elbow approaches, Total elbow replacement, Distal humerus plates

## Abstract

**Background:**

Management of distal humerus fractures remains a challenge for trauma surgeons and advancements in treatment options continue to be made to achieve the best results for patients presenting with these complex fractures. Our aim in this article is to provide the surgeons with a detailed review of current literature to help them make an evidence based decision when faced with managing such complex injuries in their surgical practice.

**Methods:**

This is a comprehensive review of the current literature that details various aspects of distal distal humerus fractures such as classification, surgical anatomy, surgical approaches, treatment options, choices of devices, outcomes and complications.

**Results:**

With the advancements in techniques and equipment, there has been improvement in patients’ outcomes following surgical management of these fractures and a large proportion of these patients are able to achieve pre-injury level of function. The contoured locking plates have enabled successful fixation of many of these fractures that were previously considered unfixable. For those not amenable to surgical fixation, total elbow arthroplasty and elbow hemiarthroplasty are considered as good alternatives.

**Conclusion:**

Since the days where the ‘bag of bones’ technique was the preferred method of treating these complex injuries, techniques and outcomes have advanced greatly. However, they still present a significant technical challenge and need meticulous technique and experience to achieve optimal results.

## 1 INTRODUCTION

Fractures of the distal humerus continue to present a significant dilemma in management despite recent advances in surgical technique. Mercifully these fractures remain uncommon with a UK incidence of 5.7 per 100,000, and constitute 2% of all fractures in adults [1].The resulting functional deficits can be profound, and the limited soft tissue envelope surrounding the elbow also means these injuries are often open.

Formerly, they were seen in young males following high energy trauma, but the last few decades have seen an increase in elderly females resulting from relatively low energy trauma. Palvenen *et al*. [2] noted a steady increase in these injuries in this patient population between 1970 and 1998. The same group [3] then noted a subsequent decline from 1998 to 2014, the reasons for which were unclear but likely were related to better functional ability of older women, as well as false prevention measures.

Distal humeral fractures in osteoporotic bone are particularly problematic due to the propensity for intraarticular comminution, poor bone stock for solid fixation as well as limited space for fixation devices. Results of internal fixation, although improved, are not without complications. Indeed total elbow arthroplasty as primary treatment is gaining popularity in selected patients [4].

In this article, we review current literature pertaining to decision making, surgical technique, complications and outcomes.

## 2 CLASSIFICATION

Many classification systems have been proposed over the years, but the more successful systems pay close attention to the number of columns (*i.e.*, medial and lateral columns) affected in addition to the articular involvement.

**Fig. (1):**
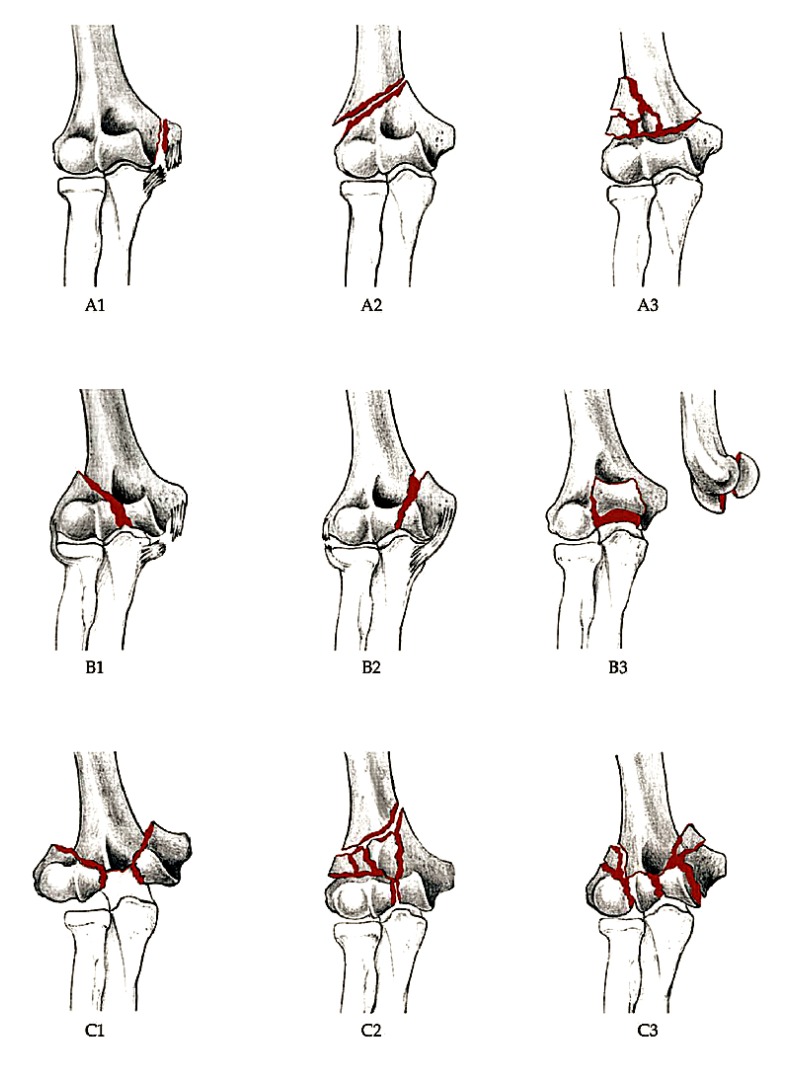
AO OTA classification of distal humerus fractures (*From Journal of orthopaedics trauma. Marsh* *et al. 2007*).

The AO/OTA classification essentially subdivides these into extra-articular (type A) fractures, intra-articular single-column (type B) fractures, and intra-articular both-column (type C) fractures. Each subtype is then further divided into location and comminution of the fracture. Articular shearing fractures of the capitellum and trochlea are classed as type B3 fractures by this classification (Fig. **1**) [5]

**Fig. (2):**
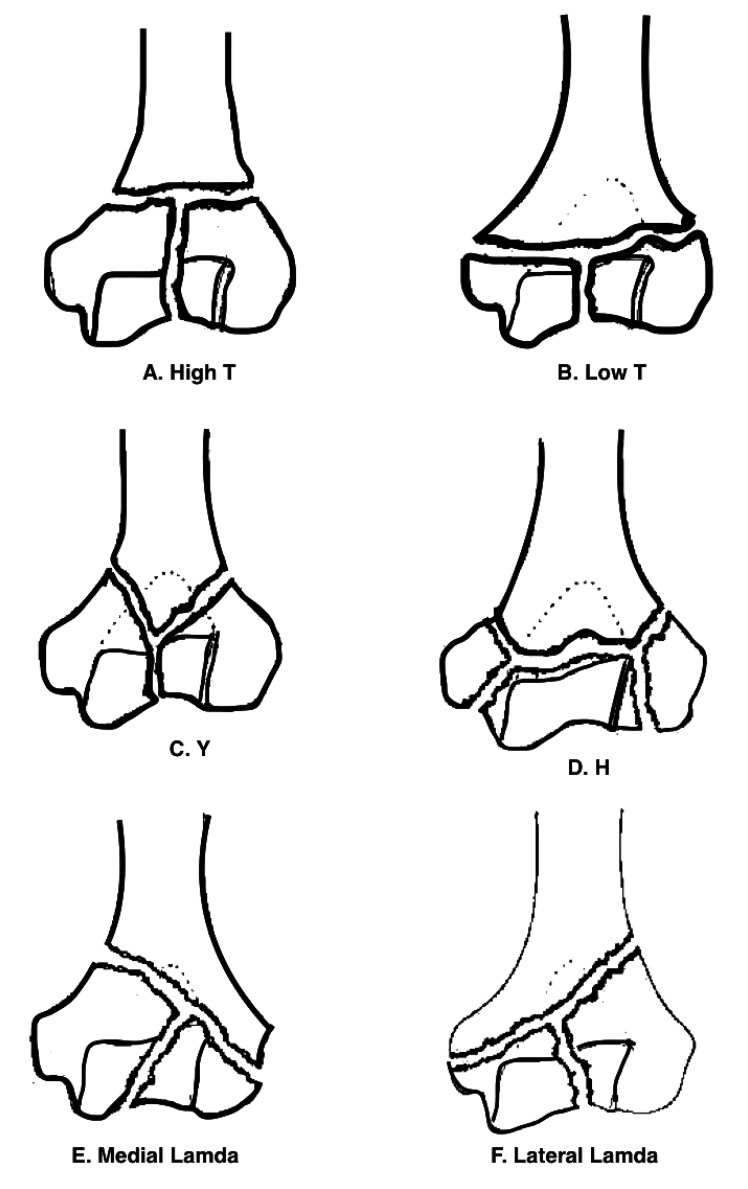
The Jupiter and Mehne classification of distal humerus fractures (*From Jupiter and Mehne. Orthopaedics, 1992*).

The Jupiter and Mehne classification of two column injuries utilises the location and slope of the fracture lines as well as the orientation of the articular fragments. In all, there are 6 main patterns in this system, comprising high and low “T”, “Y”, “H” and medial and lateral Lambda (Fig. **2**) [6]. This system is useful for predicting the success of internal fixation by the size of the constituent fragments. Low fractures and fractures of an “H” configuration often consist of many smaller fragments which are not only more difficult to reduce and hold, but may also have an increased risk of avascular necrosis [7].

## 3 SURGICAL ANATOMY

**Fig. (3):**
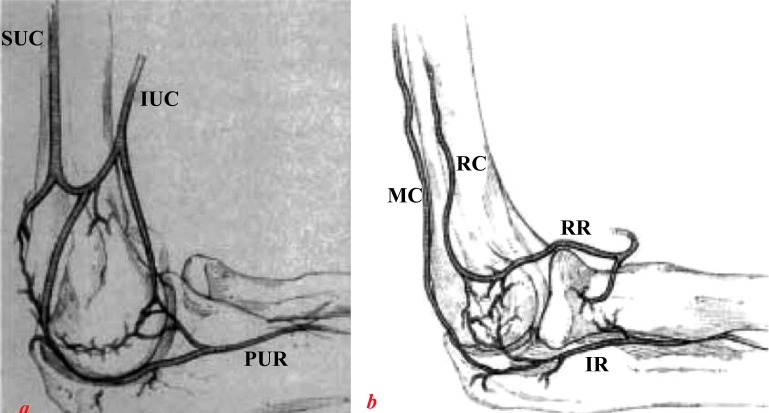
Sketches of medial (a) lateral and (b) blood supply to the distal humerus. SUC, superior ulnar collateral artery; IUC, inferior ulnar collateral artery; PUR, posterior ulnar recurrent artery; IR, interosseous recurrent artery; MC, middle collateral artery; RC, radial collateral artery; RR, radial recurrent artery. From Yamaguchi *et al*. [7].

The distal humerus consists of medial and lateral columns with an interposed trochlear. This creates a triangular structure in the frontal plane. Stable fixation of these three components is the cornerstone of obtaining a good functional result following internal fixation [8]. Of particular importance is the blood supply to this region. Two cadaveric studies [9, 10] have identified constant intra and extra osseous arterial anatomy, which they then divided into 3 arterial arcades; medial, lateral and posterior. Relative watershed areas were shown to exist in the territory between these arterial arcades- most likely the reason why low “H” configuration fractures are prone to avascular necrosis. It has likewise been shown that the lateral column receives its blood supply predominantly via posterior segmental vessels, in contrast to the medial column whose blood supply is from both anterior and posterior segmental vessels [10] (Fig. **3**). The authors state that if posterior plating is the preferred method of fixation for a fracture involving the lateral column, meticulous effort should be made to preserve periosteum, if not avoided altogether.

One of the most disastrous consequences following internal fixation for such fractures is iatrogenic neurological injury. As such the operative surgeon must have intimate knowledge of the course and relations of the adjacent nervous structures; namely the radial and ulnar nerves.

The radial nerve enters the arm through the triangular interval before giving branches to the medial head of triceps. It then comes to lie in the spiral groove of the humerus where it innervates the lateral head of triceps. From there, it pierces the lateral intramuscular septum approximately 10cm proximal to the lateral epicondyle, before passing between brachialis and brachioradialis [11]. Here, it divides into the superficial branch and the posterior interosseous nerve just anterior to the lateral epicondyle.

The ulnar nerve descends the arm on the posteromedial aspect of the brachial artery before piercing the intramuscular 8-10cm proximal to the medial epicondyle. Here it begins its journey through a fibro-osseous tunnel formed by the ligament of Osbourne, the arcade of Struthers and the two heads of flexor carpi ulnaris. Its proximity here to the medial epicondyle renders it vulnerable during fixation of distal humeral fractures, and so care should be taken to identify and protect it.

## 4 HISTORICAL OVERVIEW

Historically, distal humerus fractures had gained a reputation for universally poor outcomes regardless of treatment modality. Indeed it took many years to reach a consensus as to whether these injuries warranted surgery in favour of the non-surgical “bag of bones” technique as described by Eastwood in 1937 [12]. Zagorski and colleagues [13] were the first to prove distinct advantages of surgery versus non-operative measures. They reviewed 42 patients, of whom 29 were treated with internal fixation and the remainder treated conservatively. With an average follow up of 26 months (range 9-62 months), 76% of the surgically treated group had an excellent or good result, versus only 8% in the non-operative group.

Since then, there have been many papers looking at the prerequisites for successful fixation of these fractures. The consensus of opinion is that excellent or good outcomes follow anatomic reduction of the articular surface, restitution of the geometry of the distal humerus and stable fixation of the fragments to permit early mobilization [14-16]. Although these goals are widely accepted to be important, it may often be difficult to achieve in the presence of osteoporotic or comminuted bone [17].

### 4.1 Optimal Approach

**Fig. (4):**
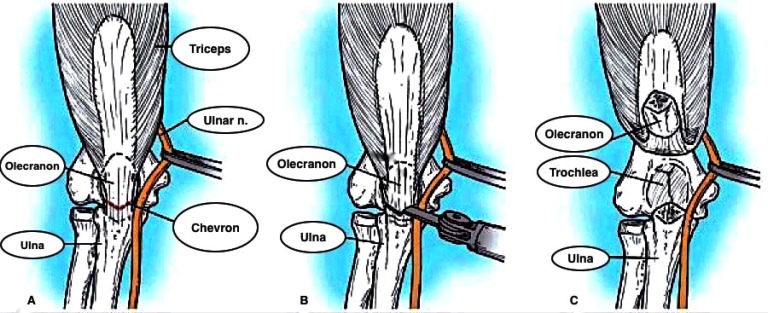
Olecranon osteotomy approach. **A**: Olecranon osteotomy is marked in shape of shallow V or chevron. **B**: Thin-blade oscillating saw is used to start osteotomy. **C**: Osteotomized proximal olecranon fragment is elevated proximally; ulnar nerve is isolated, mobilized, and protected (*From Canale & Beaty: Campbell's Operative Orthopaedics, 11^th^ edition, Mosby 2007*).

**Fig. (5):**
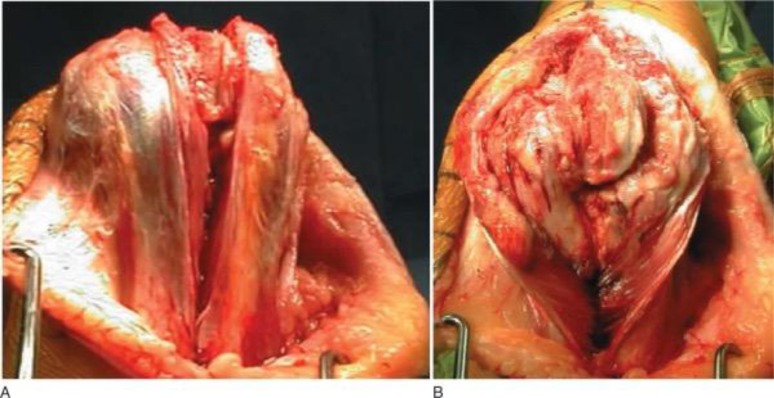
Triceps-splitting approach to distal humerus. **A**: Triceps split. **B**: Split extended to transcutaneous border of ulna. (*From Frankle MA: Triceps split technique for total elbow arthroplasty, Tech Shoulder Elbow Surg 3:23, 2002*).

**Fig. (6):**
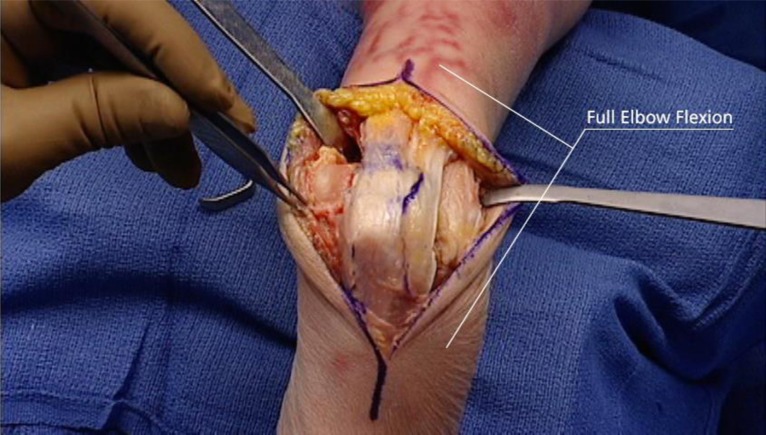
Paratricipital approach (*From Advanced Surgical Approaches to the Humerus. Depuy Synthes Institute*).

**Fig. (7):**
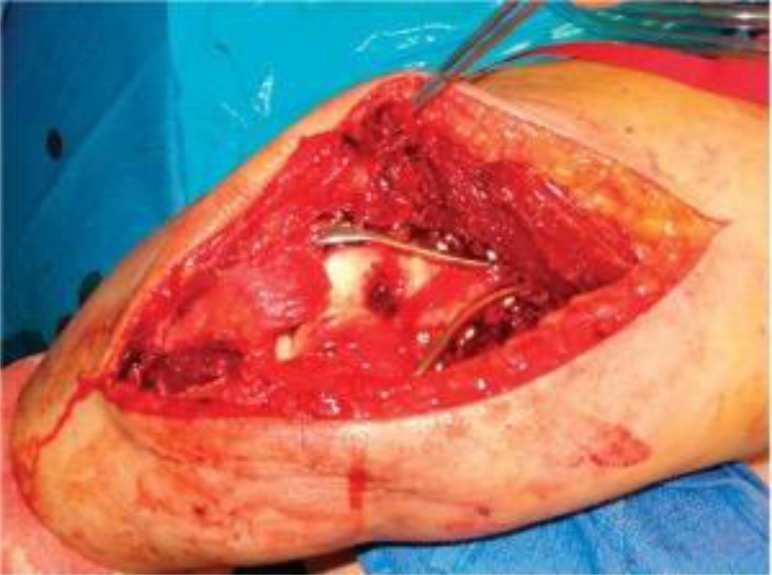
Plate application though Triceps reflecting approach to distal humerus (*From Canale & Beaty: Campbell's Operative Orthopaedics, 11^th^ edition, Mosby (2007)*).

**Fig. (8):**
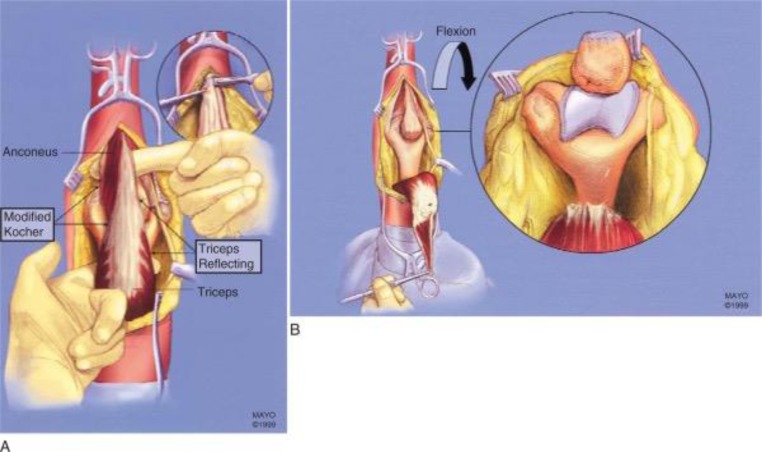
Triceps-reflecting anconeus pedicle approach. **A**: Modified Kocher lateral approach is combined with medial triceps-reflecting approach. **B**: Access to distal humerus is similar to that provided by olecranon osteotomy. (*From Sanchez-Sotelo J, Torchia ME, O'Driscoll SW: Principle-based internal fixation of distal humerus fractures, Tech Hand Upper Extremity Surg 5:179, 2001*).

Surgical opinion regarding the optimal surgical approach to distal humerus fractures remains highly divergent. Although most sources concur the straight dorsal incision is the most appropriate, the debate arises when considering how to negotiate the triceps and how best to visualise the articular surface of the distal humerus. A plethora of solutions to this have been proposed, including the olecranon osteotomy (Fig. **4**) [18], triceps-splitting exposure (Fig. **5**) [19], paratricipital exposure (Alonso-Llames) (Fig. **6**) [20], triceps sparing exposure (Bryan-Morrey) (Fig. **7**) [18] and triceps reflecting anconeous pedicle (Fig. **8**) [21].

The choice of which depends on surgeons’ preference and the surgical objectives (Table **1**).

The purported benefits of triceps sparing *versus* splitting approaches for the distal humerus are less scar formation, less blood loss and less trauma to the triceps muscle itself. It may also aid in reducing the post-operative contracture [22]. There have been relatively few studies comparing the two, and the resulting level of evidence is poor. Remia **et al**. [23] conducted a small review of 9 paediatric patients who underwent open reduction and a T-condylar distal humerus through a Bryan-Morley triceps splitting approach, and compared the results with another study [24] in which 6 distal humeral fractures in paediatric patients were treated with open reduction *via* a triceps splitting approach. They found no statistically significant difference in the outcomes between the two. Evidently, this looks at a different patient demographic than is addressed here. Likewise, the numbers were small from 2 separate surgeons. A more recent paper [25] has looked at the outcomes of open reduction for extra-articular distal humerus fractures using the triceps sparing vs splitting approach. They found a statistically significant increased range of movement and triceps strength during the rehabilitation period in the triceps-sparing group, but noted there were similar long term functional outcomes, as assessed with a DASH score. This again is not directly comparable with our patient demographic, as extra-articular fractures do not require the exposure of articular surface as do complex intra-articular injuries.

More work has been conducted comparing triceps splitting with olecranon osteotomy. Three studies found no statistically significant differences between the approaches in terms of objective elbow strength, range of motion, or functional outcomes [26-28]. One of these papers did note an increased rate of reoperation in the olecranon osteotomy group, owing to hardware removal in 27% of cases [26]. Other studies have found the rate of hardware removal to range from 6-30%, and the rate of olecranon non-union to be between 0-9% [29-32]. The evidence is somewhat stronger in support of the triceps splitting approach when dealing with open fractures. Here, one study [33] found better functional outcomes and improved range of movement when using a triceps splitting approach *vs.* olecranon osteotomy. They proposed that this was a result of the fact that most of the open fractures had already disrupted the triceps, which could be incorporated into the approach.

Zhang and colleagues [34] have also compared the olecranon osteotomy to the triceps sparing approach when dealing with type C fractures of the distal humerus. With a follow up of 6 years, they looked at 36 patients treated via an olecranon osteotomy and 31 *via* the triceps sparing approach. They observed a statistically significant reduction in procedure times, blood loss, complications rates and Mayo Elbow Performance Score (MEPS) outcomes when using the triceps sparing approach to olecranon osteotomy.

In conclusion, there is little evidence at present to support the use of triceps sparing *vs.* triceps splitting approaches in the treatment of complex distal humeral fractures. Triceps splitting may however lead to equivalent functional outcomes when compared with olecranon osteotomy, but with a lower reoperation rate for hardware removal. A triceps splitting approach is also the preferred manner of treating open distal humeral fractures.

## 5 ULNAR NERVE TRANSPOSITION

A recognised complication of distal humeral fractures is ulnar nerve injury. Controversy still exists as to whether or not ulnar nerve transposition is necessary in all cases.

One level II paper [35] looked at 29 patients with a distal humeral fracture with pre-operative ulnar nerve symptoms, and compared anterior transposition with in situ decompression. They found a statistically improved rate of complete ulnar nerve recovery (80%) in the anterior transposition group, compared with *in situ* decompression alone (57%).

Conversely, a number of studies have looked at routine anterior nerve transposition in patients who had normal preoperative ulnar nerve function. The rate of post operative ulnar neuropathy was found to be between 0-12.5% [33, 35-39]. Doornberg **et al*.* [40] analysed 30 patients treated without ulnar nerve decompression, with a follow up between 12 and 30 years. Only one patient had signs of ulnar nerve dysfunction at the final follow up. They therefore concluded that routine ulnar nerve decompression was not routinely indicated.

And so, there is currently insufficient evidence to support the routine ulnar nerve transposition in all distal humeral fractures, it would appear to be beneficial to those who have preoperative symptoms.

To investigate it further, an RCT titled “A Multicentre, Randomized Trial of Simple Decompression *Versus* Anterior Transposition of the Ulnar Nerve for Acute, Displaced Fractures of the Distal Humerus Treated With Plate Fixation” is currently on going in Toronto, Canada. The results from this study are currently awaited [41].

## 6 CHOICE OF DEVICE

Having established the benefits of internal fixation in distal humeral fractures, attention was then turned to the most appropriate choice of implant. Dynamic compression plates (DCP) quickly fell out of favour owing to their bulk and difficulty in contouring. 1/3 tubular plates, by contrast, were easier to contour and were sufficiently low profile, but did not confer sufficient stability. Indeed Henley **et al.** [29] found a 15% rate of hardware failure with their use in treating distal humeral fractures. They therefore recommended against their use.

The advent of the locking plate brought renewed interest into this topic. Korner **et al.** [42] conducted a biomechanical study that concluded locking plates provided increased primary stiffness to both anterior/posterior bending and torsional loading when compared with conventional reconstruction plates. O’Driscoll and colleagues [17] concurred with this finding.

Most recently, locking plates precontoured to the distal humerus have become increasingly popular. Schuster **et al.** [43] performed a cadaveric study comparing conventional reconstruction plates, locking compression plates, and locking plates pre contoured to the distal humerus. All cadavers were matched for fracture configuration and were divided into two groups by bone mineral density, determined using qualitative CT scanning. There was no significant difference between choice of plate in the good bone mineral density group. However in the poor bone mineral density group, the locking plates provided improved resistance to screw loosening than the non-locked constructs. Further to this, the failure rate was significantly lower in the distal humerus plates than with locking compression plates. They therefore concluded the use of distal humerus plates to be advantageous in the treatment of these injuries in osteoporotic bone.

This has since been confirmed in the literature, with Gupta *et al.* [44] finding very low implant failure rate with the use of precontoured, anatomical locking plates in distal humeral fractures in the elderly population.

## 7 PLATE CONFIGURATION

Previous doctrine had stated that the optimum method of fixation of two column injuries was by way of two plates orientated at 90º to one another [14, 45, 46]. The medial column was typically plated medially with the lateral column plated posteriorly- the “90º/90º” technique. No study however, conclusively proved that this construct conferred any more stability than parallel plating, and likely emanated from an era where reconstruction plates were significantly less strong.

O’Driscoll [16] noted that the lateral column was often the first to fail as a result of excessive varus forces acting on the elbow during normal activities of daily living. He asserted that a posterior plate would confer less resistance to varus forces than would a parallel plating technique. This was confirmed in a biomechanical study by Self **et al.** [46] who showed that failure of 90º/90º plates occurred with screws pulling out of the lateral column distally. They also demonstrated that plating in the sagittal plane conferred greater stiffness than the 90º/90º technique. Another problem with posterior plating for the lateral column results from the smaller anteroposterior diameter of the humerus, permitting only one or two short screws for fixation. This is in contrast to the sagittal plane, where longer screws can be accommodated. As shown by Kimball **et al.** [10], the majority of the blood supply to the lateral column is also derived from posterior segmental vessels. Sagittal plane plating has less risk of injuring these structures, which may improve chances of union.

The parallel plate fixation strategy focuses on maximising stability between the distal fragments and the shaft of the humerus at the metaphyseal level. According to O’Driscoll, this can be achieved by following a set of eight technical objectives: (1) Every screw in the distal fragment should pass through a plate. (2) Every screw in the distal fragment should be anchored in a fragment on the opposite side that is fixed by a plate. (3) As many screws as possible should be placed in the distal fragment. (4) Every screw in the distal fragment should be as long as possible. (5) Every screw in the distal fragment should engage as many articular fragment as possible. (6) The screws in the distal fragments should lock together by interdigitation, creating a fixed angle structure, thereby completing the arch or closing the loop. (7) The plate should be applied with compression at the supracondylar level. (8) The plate should be strong and stiff enough to resist bending or breakage [17].

O’Driscoll also advocates the use of two plates are of differing lengths to prevent the formation of a stress riser. This is purported to reduce the risk of periprosthetic fracture.

A study by Lan **et al*.* [47], however, showed that both perpendicular and parallel locked plate configurations with the appropriate surgical techniques can provide anatomical reconstruction and stable fixation of type C intra-articular distal humeral fractures and allow early mobilisation of the elbow. There were no significant differences in the surgical time, blood loss, bone union time Mayo Elbow Performance Score (MEPS), flexion-extension arc and the total range of flexion and extension between the two groups.

Although parallel plating has become more popular of late, both methods are still regarded as acceptable techniques in the treatment of these injuries.

## 8 THE ROLE OF TOTAL ELBOW ARTHROPLASTY (TEA)

Comminuted, intra-articular distal humeral fractures present a significant challenge even in young patients with adequate bone stock. Osteoporotic bone has a propensity for significant comminution whilst concurrently affording poor fixation. So much so, that this may render the challenges faced during ORIF insurmountable. As a result, there has been a recent move to support the use of primary TEA in the treatment of this patient group.

McKee and colleagues [48] conducted a prospective, randomised, multicentre controlled trial to compare open reduction (21 patients) with primary semiconstrained TEA (21 patients) in complex, intra articular distal humeral fractures in patients over the age of 65. They used reoperation rate as the primary outcome measure, and a Mayo Elbow Performance Score (MEPS) as a secondary outcome measure. Although 42 patients were included in the study, 2 patients did not complete the study, and there were 5 patients requiring conversion from ORIF to TEA. This left 15 patients in the ORIF group and 25 in the TEA group. Even considering this, they found no significant difference in the rate of reoperation, they did find that the TEA group had significantly improved MEPS at 3 months, 6 months, 12 months and 2 years in comparison to those treated with ORIF. Although they did find a slightly improved range of motion in the TEA group, this was not significantly significant at the 2 year mark.

A more recent study [49] looked at 87 patients over the age of 65 with a distal humeral fracture treated with TEA. This included all classifications of distal humeral fractures, including type A (9 cases) and type B (8 cases), but the majority were type C in 70 cases. With a mean follow up of 37.5 months, the mean MEPS was 86 and the mean DASH was 24. 63% of patients had a pain free elbow. 48% of patients had a mean flexion-extension arc of at least 100º, with normal function obtained in 79% of patients. Repeat surgery was required in 8 cases (9%) for a variety of indications **i.e.*,* stiffness, ulnar nerve decompression *etc*. This also included two revisions, one for periprosthetic fracture and one for aseptic loosening.

These results were similar to report from Frankle **et al.** [19]. They reviewed the results of 24 females over the age of 65 treated with ORIF (12) and primary TEA (12). At 2 year follow up, the MEPS for TEA group was 11 excellent and 1 good. This contrasted to the ORIF group with 4 excellent, 4 good, 1 fair and 3 poor. This study was not without criticism, largely owing to the small numbers and the fact that they did not exclude rheumatoid patients. It was underpowered and so no statistically significant conclusion could be made.

Garcia and colleagues [50] also conducted a review of 19 patients treated with TEA for distal humeral fractures between 1995 and 2000. All patients were over the age of 60. 2 patients died and 1 was excluded due to dementia. With a mean followup of 3 years, 68% reported having no pain, with a mean flexion arc of 24º-125º. The mean DASH score was 23 and the mean MEPS was 93. 15 implants were radiographically well fixed at final followup. A single patient had developed a radiolucent line at the cement bone interface, but this was present on the initial post operative film and was not progressive.

## 9 TEA FOLLOWING FAILED ORIF

Having established the role of primary TEA in the treatment of these fractures, many surgeons still prefer to attempt ORIF in a bid to avoid the restrictions and long term complications associated with the elbow arthroplasty; not to mention the possibility of revision surgery. In doing so, they accept the risk that secondary revision to TEA may be necessary in the event of failed fixation. Fortunately, the current evidence supports this as a viable treatment option, with results of secondary TEA comparable to that of primary TEA.

Prasad **et al.** [51] studied 32 distal humerus fractures, with 15 treated with primary TEA and 17 treated with ORIF with subsequent conversation to TEA. The mean followup was 56.1 months. The MEPS was not significantly different between the two, with 84% in the primary TEA group obtaining good or excellent results, compared with 79% in the delayed group. Subject satisfaction was 92% in both groups. There was a slightly higher rate of complications in the delayed group, with 2 cases of infection, 2 ulnar nerve palsies, 1 case of heterotrophic ossification and 1 case of aseptic loosening, compared with 1 case of complex regional pain syndrome and three cases of aseptic loosening in the primary TEA group. Likewise, there was no difference in survivorship by Kaplan Meier analysis. Overall, they found no statistically significant difference between the two groups.

In summary, the evidence supports the use of TEA, either primary or delayed, as a valid treatment option to complex, comminuted distal humeral fractures in the elderly. In younger patients, however, every effort should still be made to preserve the native joint as their life expectancy outlasts the implant longevity.

## 10 THE ROLE OF ELBOW HEMIARTHROPLASTY (EHA)

Undoubtedly good functional results can be achieved with TEA for complex fracture patterns involving the distal humerus. The disadvantage with this approach is options for revision are somewhat limited should it fail. Aseptic loosening, polyethylene wear, osteolysis and periprosthetic fracture are all documented complications [26], and this has led some surgeons to consider EHA as a possible alternative to keep the option for later conversion to TEA open.

Three main prosthesis have contemporary published data in the literature; the Kudo (Biomet Ltd, Brigend, UK), the Sorbie-Questor (Wright Medical Technology, Arlington, TNUSA) and the Latitude (Tornier, Montbonnot-Saint-Martin, France). The Kudo is a non anatomic implant, which has a proven track record as an unlinked TEA prosthesis. The Sorbie-Questor is anatomical, but is no longer available. The Latitude is an anatomic, modular prosthesis which facilitates conversion to either linked or unlinked TEA. This makes it seem ideal for use in this role.

Given the unconstrained design philosophy of these implants, restoration of the condyles and collateral ligament complex is essential in ensuring stability of the joint.

A recent review of current literature by Phadnis **et al.** [52] analysed 121 reported cases in 7 papers [53-59] of EHA being utilised for distal humeral fractures, with a mean follow up of 37.5 months. The mean MEPS was 87.6 (SD 14.5), with outcome being classed as excellent or good in 86% of patients. The authors comment the trans-olecranon approach correlated with significantly worse MEPS, and there was also a non significant trend to worse MEPS with younger age. Likewise the mean quick-DASH (94 patients) was 18.3. These results compare favourably with those for TEA for distal humeral fractures.

Surgical complications reached 18%-chief of which was ulnar nerve irritation in 11 patients, followed by peri-prosthetic fracture (5 patients) and infection (2 patients). The rate of reoperation was 28% for removal of metal work in 14 patients, ulnar nerve decompression (6 patients) and revision to TEA (5 patients). The complication rate is comparable to that of TEA for trauma. Interestingly, only 1 case was revised for instability, necessitating a lateral collateral ligament reconstruction. The authors of the review suspect this may however be under reported.

When looking solely at revision of EHA to TEA, the authors found a non-significant number had been performed for post-traumatic salvage, and so caution its use in such cases. They also recommend against using the trans-olecranon approach and its use in young patients for trauma. They conclude by recommending its use in active, elderly patients with an acute fracture.

## 11 OUTCOMES

With the advances in technique and equipment, we have seen improvements in patient outcomes following ORIF for complex distal humeral fractures. Approximately 70% of patients achieve satisfactory outcomes with a functional arc of more than 100º. Mean post-operative flexion arcs appear to be between 97º-112º, and supination arcs between 151º-165º^.^ The resulting strength in the operated arm appears to be good, with patients achieving around 75% of the strength of the uninjured side [38, 39, 46, 60, 61], allowing three quarters of patients to return to a level of function similar to their pre-jury status [13]. Patient satisfaction has been reported to be as high as 85-95% [32, 62].

Kaiser **et al.** [61] looked at 22 distal humeral fractures treated using 2 orthogonal distal humeral plates. At a mean follow up of 30.5 months, all fractures had united and achieved a mean range of motion of 129º flexion and -16º extension. No secondary displacement of the fracture was observed.

The results are similar to those obtained by Erpelding **et al.** [63], who looked at 37 distal humeral fractures treated by internal fixation through the paratricipital approach. The mean range of motion was 126º with excellent DASH scores. The triceps strength was 90% that of the uninjured side.

## 12 YOUNGER PATIENTS

Whilst we have seen that TEA leads to predictably good results following distal humeral fractures in the elderly population, there still remain concerns surrounding its use in young patients because of concerns regarding implant loosening and clinical failure. Park **et al.** [64] recently performed a followup of 23 TER's in patients with a mean age of 33 years (range 20 - 43 years). The main diagnosis was post traumatic arthritis and non traumatic arthritis. They found good improvements in pain and function, with a 15 year survivorship of 89%.

In spite of these positive findings, the implant longevity is still likely to be less than the life expectancy of younger patients. And a failed total elbow arthoplasty leaves few avenues to explore. For that reason, most surgeons would opt for suboptimal fixation and preservation of the patients native joint, than proceed directly to TEA in such a patient demographic. There is little to be lost with this approach, as there is no statistically significant differences in outcomes between primary and delayed TEA for distal humeral fractures.

The question then arises in what to do in terms of rehab for the young patient with tenuous fixation of a distal humerus fractures. In this situation, it is probably sensible to opt for slow rehab to preserve the natural anatomy, whilst accepting that stiffness will be a problem at a later date. Indeed, a recent paper from Koh **et al.** [65] suggests that an acceptable movement arc of 100° can be obtained following open arthrolysis of stiffness following distal humeral fracture fixation. That said, there is little evidence in the literature pertaining to these complex injuries in the younger patient group.

## 13 COMPLICATIONS

There are a number of complications associated with Open Reduction and Internal Fixation (ORIF) for these fractures. Of these, the most common are non-union, heterotrophic ossification, ulnar nerve neuropathy and deep seated infection.

Ulnar neuropathy is perhaps one of the most common complications following surgical fixation. Most papers have found the risk of ulnar neuropathy to be between 0-15% [9, 43, 66] with one paper finding it to be as high as 38% [67]. Risk factors for ulnar neuropathy include pre-existing ulnar nerve dysfunction, excessive traction, impingement on the implant, and excessive scar formation [8]. McKee and colleagues [68] looked at the role of ulnar nerve neurolysis for post traumatic elbow reconstruction. They found significantly improvements in grip strength, lateral strength and tip pinch strength along with high rates of patient satisfaction. There is however no strong evidence to support the use of routine ulnar nerve transposition, providing there are no pre-operative symptoms (discussed in detail already under the ulnar nerve transposition heading).

The rate of non union and implant failure is between 0-13% [27, 37, 62, 69] and is frequently the result of unsatisfactory fixation at the time of the index procedure. Ali *et al.* concluded this was the cause in over 75% of cases [70]. In other cases, high energy trauma, high comminution and poor bone stock was cited as reasons for failure. The risk of non union is also reportedly higher in low configurations of distal humeral fractures [66]. Revision surgery for non union is a technically challenging procedure, fraught with risk and complication. If fixation is not possible, very unstable, or resistant to healing, external fixation, fibular strut grafts or total elbow arthroplasty may be considered as viable alternatives [45, 71].

Heterotopic Ossifiation (HO) can cause significant reduction in range of movement and outcome. Risk factors include concomitant central nervous system injury, delay in surgical intervention, and open fractures. Pooled analysis of data from a number of studies shows on overall prevalence of 8.6% [37, 72, 73]. Shin **et al.** [38] reviewed 35 patients undergoing surgical fixation for distal humeral fractures, and administered one dose of radiotherapy on the first postoperative day, followed by a two week course of indomethacin. The found the rate of symptomatic HO to be 2.9%, with a non union rate of 5.7%. Liu **et al.** [74] looked at 32 patients treated with a 6 week course of celecoxib. Here they found a 3.1% rate of symptomatic HO, with 0% non union. This said, the results were too underpowered to achieve statistical significance and to recommend the routine use of prophylaxis when treating distal humeral fractures. Some surgeons would still consider its use in high risk individuals.

The rate of deep seated infection ranges from 0-8% [27, 32, 37, 60, 69]. The cornerstone of management lies in surgical debridement and antibiotic therapy as guided by tissue cultures. If the fixation is deemed to be stable, then the patient may be treated with repeated debridement and antibiotics until healing is evident. If stability is compromised, a staged revision may be required; be it revision ORIF or conversion to TEA.

## CONCLUSION

Current concepts and techniques have progressed dramatically since the days where the ‘bag of bones’ technique [12] was the preferred method of treating these complex injuries. With advancing experience, we have seen improved outcomes and patient satisfaction. The key to obtaining good post operative function lies in anatomical reduction of the articular surface and good compression between the two columns. Stable fixation and early mobilisation is the goal, and if these criteria are satisfied, there is much literature to suggest good outcomes will follow.

Given the highly complex nature of these injuries, we fully realise that fixation is not always possible. In such an eventuality, these injuries may be treated perfectly reasonably by means of TEA which has good level 1 studies to support its use in the literature. Moreover, EHA may be used to produce good effect in acute fractures in active elderly patients.

In spite of the wealth of literature now surrounding the management of these injuries, they still present a significant technical challenge- even to the most seasoned of surgeons. Complex fracture patterns, variable soft tissue envelopes, and poor bone stock mean these are not injuries for the “occasional surgeon”. Whilst we commend to the reader the use of the above techniques in the treatment of such injuries, it should not be used as a substitute for previous experience of treating these injuries. Experience in itself is likely to be the major determinant of success. We therefore advocate the involvement of a surgeon with a special interest in these injuries at an early stage.

## Figures and Tables

**Table 1:** Surgical Approaches Used for Treatment of Fractures of the Distal Humerus (Canale & Beaty: Campbell's Operative Orthopaedics, 11^th^ edition, Mosby (2007)17.

**Surgical Approach**	**Indications**	**Contraindications**	**Advantages**	**Disadvantages**
Olecranon osteotomy	Open reduction and internal fixation (ORIF) for fractures involving columns and articular surface	Total elbow replacement (TER)	Good access to posterior articular surfaces for reconstruction	Nonunion and failure of fixation of osteotomy Poor anterior access to capitellum
Triceps-splitting	ORIF/TER for fractures involving columns and articular surface	Previous olecranon osteotomy approach Patients at increased risk for healing problems	Avoids complications associated with olecranon osteotomy	Poor access to articular surface for internal fixation Risk of triceps detachment
Triceps-reflecting	Fractures requiring TER	ORIF Previous olecranon osteotomy approach Patients at risk for healing problems	Avoids complications associated with olecranon osteotomy	Risk of triceps detachment
Triceps-detaching	ORIF/TER for fractures involving columns and articular surface	Previous olecranon osteotomy approach Patients at risk for healing problems	Avoids complications associated with olecranon osteotomy	Poor access to articular surfaces for internal fixation Risk of triceps detachment

## LIST OF ABBREVIATIONS

DASH: Disabilities of the Arm, Shoulder and Hand
EHA: Elbow Hemiarthroplasty
HO: Heterotopic Ossification
MEPS: Mayo Elbow Performance Score
ORIF: Open Reduction and Internal Fixation
TEA: Total Elbow Arthroplasty
TER: Total Elbow Replacement
